# Clade 2.3.4.4b H5N1 HPAIV from Migratory Birds in Beidaihe Wetland, North China

**DOI:** 10.3390/v18060595

**Published:** 2026-05-25

**Authors:** Yiyang Zhang, Xiaoli Bai, Chenhui Nie, Yufei Guo, Chao Shan, Yanxia Xiao, Xiaoqing Zhang, Shuaiyu Jiang, Yongmei Su, Cheng Chang, Yongsheng Liu, Shunli Yang, Yanbing Li, Jie Tian, Boru Zhang, Bin Liang, Alexei D. Zaberezhny, Yunkai Qian, Jie Zhang, Xiaorui Zhang

**Affiliations:** 1 Hebei Province Key Laboratory of Preventive Veterinary Medicine, College of Animal Science and Technology, Hebei Normal University of Science and Technology, Qinhuangdao 066004, China; 17636267050@163.com (Y.Z.);; 2 State Key Laboratory for Animal Disease Control and Prevention, Harbin Veterinary Research Institute, Chinese Academy of Agricultural Sciences, Harbin 150069, China; xiaolibai_98@163.com (X.B.);; 3 Qinhuangdao Bureau of Agriculture and Rural Affairs, Qinhuangdao 066001, China; 4 Qinhuangdao Customs Technology Center, Qinhuangdao 066099, China; 5 Key Laboratory of Special Animal Epidemic Disease, Ministry of Agriculture and Rural Affairs, Jilin Provincial International Cooperation Key Laboratory for Science and Technology Innovation of Special Animal and Plants, Institute of Special Animal and Plant Sciences, Chinese Academy of Agricultural, Changchun 130112, China; 6 The Science and Technology Research Center of China Customs, Beijing 100026, China; 7 Qinhuangdao Forestry Bureau, Qinhuangdao 066001, China; 8 Federal State Budgetary Institution All-Russian Research and Technological Institute of Biological Industry (VNITIBP),17,bldg.1,Village of the Biokombinat, Losino-Petrovsky Urban District, Moscow Oblast 141142, Russia

**Keywords:** highly pathogenic avian influenza virus (HPAIV), clade 2.3.4.4b H5N1, migratory birds, Beidaihe Wetland, pathogenicity, antigenicity

## Abstract

During 2022–2024, a highly pathogenic avian influenza virus (HPAIV) H5N1 strain, designated A/Seagull/Hebei/qhd6/2024 (H5N1), was isolated from migratory birds in Beidaihe National Wetland Park, North China. Phylogenetic analyses revealed that its hemagglutinin (HA) gene belongs to the 2.3.4.4b clade, while the neuraminidase (NA) gene and internal genes clustered with strains originating from multiple continents, consistent with a transcontinental reassortment event. The virus also exhibited 90.1–98.1% nucleotide homology with human-derived H5N1 isolates. Molecular characterization identified key virulence-associated mutations, including the classic HPAIV HA cleavage site, HA-T160A (associated with enhanced human receptor-binding capacity), and NA-I117T (potentially linked to drug resistance). BALB/c mouse infection experiments confirmed systemic replication and high pathogenicity of strain qhd6, with a 50% lethal dose (LD50) of 0.95 log_10_EID_50_/mL. Antigenic analysis revealed good cross-reactivity with the widely used H5-Re14 vaccine strain. This study reports the identification, in Beidaihe National Wetland Park, of an HPAIV H5N1 strain whose genetic characteristics suggest intercontinental reassortment and indicate cross-species transmission risk. It clarifies the genetic characteristics and pathogenicity of this strain, providing an important theoretical and practical basis for precise surveillance, risk early warning, and comprehensive prevention and control of AIV at migratory bird stopover sites in North China.

## 1. Introduction

Migratory birds are integral to ecosystems. Undertaking annual long-distance intercontinental migrations along fixed flyways, their high mobility and frequent interspecific interactions make them important natural vectors for zoonotic pathogens [1,2,3,4]. These pathogens are excreted in large quantities via migratory bird feces, contaminating water, foraging grounds, and poultry farms along migration routes, posing a persistent risk to livestock, poultry breeding, and human public health [5]. Additionally, migratory birds’ gregarious behavior during stopover, breeding, and overwintering exacerbates intraspecific pathogen transmission, while their interspecific contact with wild animals and poultry in shared habitats further drives cross-species spillover and adaptive evolution of these pathogens [6,7,8].

Among zoonotic pathogens carried by migratory birds, avian influenza virus (AIV) is one of the most studied and high-profile. Based on their pathogenicity in chickens, avian influenza viruses (AIVs) are classified into low-pathogenic avian influenza viruses (LPAIV) and highly pathogenic avian influenza viruses (HPAIV). LPAIVs typically cause mild or asymptomatic infections in natural hosts, whereas HPAIVs (primarily involving certain strains of H5 and H7 subtypes) can induce systemic disease with high mortality in wild birds, poultry, and even humans [9]. In 1996, an H5N1 HPAIV was first isolated from a domestic goose in Guangdong, China (A/goose/Guangdong/1/96, termed the Gs/GD lineage). This lineage subsequently spread rapidly across Eurasia, causing multiple large-scale outbreaks worldwide and sporadic human infections. Among the continuous evolution of the Gs/GD lineage since 1996, only a few clades—clades 2.2, 2.3.2.1, and 2.3.4.4b—have demonstrated the ability to spread globally [10]. The 2.3.4.4b clade has attracted particular concern.

AIV is a negative-sense, single-stranded, segmented RNA virus of the Orthomyxoviridae family; its segmented genome makes it highly prone to mutation and reassortment when different strains co-infect a host. This continuous variation generates new variants with altered pathogenicity, transmissibility, and host range, posing major and persistent challenges for global avian influenza prevention and control [11,12]. Studies confirm migratory birds are natural reservoirs and long-distance vectors for AIV, playing a pivotal role in its global transmission [13,14]. Notably, the 2.3.4.4b clade H5N1 AIV has crossed species to infect mammals in Europe and the U.S. (e.g., foxes, otters, harbor seals), and the 2024 U.S. dairy cattle H5N1 outbreak is hypothesized to be migratory bird-transmitted [15,16], highlighting migratory birds’ critical role in AIV spread.

Of the eight global migratory bird flyways, three traverse China, making it a key node in the global migratory bird-borne pathogen transmission network. Beidaihe National Wetland Park, located in Qinhuangdao—a famous coastal tourist city along the Bohai Sea in North China—is a core stopover and wintering site on the East Asia-Australasia Flyway (EAAF) that supports a notably complex ecological structure and sustains intense migratory activity, with flyway connections spanning 22 countries and regions. Close interaction between wild migratory birds, local poultry, and human activities here increases the risk of zoonotic pathogen transmission and spillover. Given the risk of clade 2.3.4.4b dissemination along migratory flyways, systematic virological surveillance at key habitats along the EAAF, a major migratory bird route, holds indispensable value for early warning and risk assessment.

Notably, despite the Chinese government’s great emphasis on bird-born virological surveillance in Beidaihe Wetland and continuous monitoring enhancement, this study achieved a key achievement: we conducted systematic, continuous AIV surveillance of migratory birds from 2022 to 2024 in Beidaihe Wetland Park (a core EAAF stopover site) and isolated a clade 2.3.4.4b H5N1 strain from *Larus cachinnans*, a dominant local migratory waterfowl. Through comprehensive phylogenetic analysis, recombination detection, and biological characterization, we clarified the strain’s genetic variation, evolutionary origin, transmission dynamics, and cross-species transmission potential. Our findings fill the data gap in migratory bird pathogen surveillance in Beidaihe Wetland Park, and provide important theoretical and practical support for EAAF avian influenza early warning, prevention, and cross-regional joint control strategy formulation.

## 2. Materials and Methods

### 2.1. Ethics Statements and Facility

All experimental procedures related to HPAIV, including sample collection, virus isolation, and mouse experiments, were conducted in strict accordance with the standards and guidelines established by Harbin Veterinary Research Institute (HVRI), Chinese Academy of Agricultural Sciences, as well as the provisions of the “Regulations on the Administration of Biosafety in Pathogenic Microorganism Laboratories”. HVRI serves as the National Reference Laboratory for Avian Influenza in China and is also recognized as the Reference Laboratory for Avian Influenza by the World Organization for Animal Health (WOAH). The study was approved by the Institutional Animal Care and Use Committee (IACUC) of HVRI (No. 250915-04-GJ) and all operations strictly followed the principles of animal welfare and biosafety.

### 2.2. Epidemiological Surveillance of AIV in Migratory Birds in Beidaihe National Wetland Park

Sample collection was approved by relevant forestry authorities and conducted by professionals. Birds were sampled from rescued individuals at Yizhan Bird Rescue Center (Beidaihe District, Qinhuangdao) or captured by mist nets in local wetlands. Close cooperation with the center ensured birds’ welfare, and all live birds were released immediately after sampling.

Samples included oropharyngeal/cloacal swabs, fecal samples, and carcasses of wild birds found dead in the field. Swabs were placed in disposable viral transport medium tubes (Shengxia Biotechnology Co., Ltd., Shijiazhuang, China). Carcasses were stored in sealed refrigerated containers and transported to the laboratory for necropsy. All samples and dissected tissues were stored at −80 °C for subsequent analysis. A total of 1628 samples were collected and tested for AIV from 2022 to 2024.

Viral RNA was extracted using kits from Beijing Tiangen Biochemistry and Technology Co., Ltd. (Beijing, China) following the manual. AIV was detected with commercial fluorescence diagnostic kits (Guangzhou Vibroxin Biotechnology Co., Ltd., Guangzhou, China). AIV typing primers were synthesized, and PCR was performed referring to the national standard GB/T 18936-2020 [17]. PCR products were sequenced by Tianjin Kinko Biological Company (Tianjin, China).

### 2.3. Necropsy and Virus Isolation with Chicken Embryos

Under sterile conditions, post-mortem examination was performed on dead seagulls. Tissue samples including lung, liver, intestine, heart and pancreas were collected, and the gross lesions of these organs were recorded. Nucleic acids were extracted from the corresponding tissue samples, and the viral load was determined using an avian influenza fluorescence detection kit (Guangzhou ViboXin Biotechnology Co., Ltd., Guangzhou, China). Avian influenza-positive tissue samples were homogenized in sterile PBS. The homogenate was centrifuged at 12,000 rpm for 3 min, and the supernatant was collected and filtered through a 0.22 µm filter to eliminate bacterial contamination. Penicillin/streptomycin was added, and the filtrate was serially diluted 10^−1^ to 10^−5^ in sterile saline. Each dilution was inoculated into the allantoic cavity of 9–11-day-old SPF chicken embryos (Beijing Boehringer Ingelheim Viton Biotechnology Co., Ltd., Beijing, China), with three embryos per dilution. Embryos dying within 24 h were discarded. The remaining embryos were incubated at 37 °C for 72 h. Harvested allantoic fluid was centrifuged at 8000 rpm for 10 min at 4 °C to eliminate impurities, and viral titer was quantified via EID_50_ assay before storage at −80 °C.

### 2.4. Whole Genome Sequencing and Phylogenetic Tree Construction

Viral RNA was extracted from positive allantoic fluid of embryonated chicken eggs and reverse-transcribed into cDNA using the PrimeScript™ II 1st Strand cDNA Synthesis Kit (Takara, Tokyo, Japan). Second-strand cDNA was synthesized using DNA Polymerase I (TAKARA) and T4 DNA Polymerase (TAKARA). Library construction and sequencing were performed using the Illumina NovaSeq 6000 platform (Illumina, San Diego, CA, USA) with the corresponding library preparation kit. The sequencing data were quality-controlled using Fastp. Genome assembly was carried out with SPAdes and Trinity, and gene prediction was performed using GeneMarkS. The genome was sequenced at a depth of 289×. The sequencing was performed by Shanghai Winnerbio Technology Co., Ltd. (Shanghai, China).

Phylogenetic trees for the eight gene segments of avian influenza viruses were constructed with the Maximum likelihood (ML) method in MEGA 12.0 software, with 1000 bootstrap replicates to evaluate node reliability. The best-fit nucleotide substitution model was determined using IQ-TREE 2.0 software, and a maximum-likelihood phylogenetic tree was subsequently reconstructed for the HA gene segment of 140 avian influenza viruses. Annotation and graphical refinement of the resulting tree were conducted using the Chiplot online platform. Reference sequences were obtained from the NCBI and GISAID influenza databases (Table S2).

### 2.5. Replication and Virulence of H5N1 Virus in Mice

Four to six-week-old female specific-pathogen-free (SPF) BALB/c mice (15–16 g) were obtained from the BEIJING CHARLES RIVER Co., Ltd. (Beijing, China). All animals were acclimatized for three days in a biosafety level 3 (BSL-3) laboratory prior to experimentation.

To determine the 50% mouse lethal dose (MLD_50_) for virulence, 35 mice were randomly divided into six experimental groups and one control group (*n* = 5 per group). Mice in the experimental groups were intranasally inoculated with 50 μL of 10-fold serial dilutions of the H5N1 virus (10^−1^ to 10^−6^ EID_50_) under ether anesthesia, while the control group received 50 μL of sterile phosphate-buffered saline (PBS). Animals were monitored daily for 14 days post-inoculation for clinical signs, body weight changes, and mortality. The MLD_50_ was calculated using the Reed–Muench method.

Viral replication was evaluated using a separate cohort of BALB/c mice, which were randomly assigned to experimental and control groups (*n* = 3 per group). Mice were intranasally inoculated with 50 μL of H5N1 virus at a dose of 10^−6^ EID_50_, as determined by preliminary experiments, or with sterile PBS as a control. At 3 days post-infection (dpi), mice were euthanized by cervical dislocation, and major organs, including the brain, liver, spleen, nasal turbinates and lungs were aseptically harvested.

Tissues were homogenized in sterile PBS at a ratio of 1:9 (*w*/*v*) and centrifuged at 12,000 rpm for 10 min at 4 °C. Viral titers in the clarified supernatants were determined using the EID_50_ assay to evaluate viral replication kinetics and tissue tropism. The result was presented by GraphPad Prism 8.

### 2.6. Antigenic Analysis

Antigenic analysis was performed using hemagglutination inhibition (HI) assay with 1% chicken erythrocytes. For this assay, antigens from the H5-Re11, H5-Re13, and H5-Re14 vaccine strains, along with their corresponding antisera (Guosheng Biotechnology Co., Ltd., Harbin, China), were used for viral antigenic identification. The result was presented by R Language 4.6.0.

## 3. Results

### 3.1. Epidemiological Surveillance of AIV in Migratory Birds in Beidaihe National Wetland Park

From 2022 to 2024, our research team conducted systematic epidemiological surveillance for AIV in migratory birds at Beidaihe Wetland Park, Qinhuangdao City, North China. During this period, a total of 1628 migratory bird samples were collected, including 1532 pharyngeal/anal (oropharyngeal/cloacal) swabs, 81 carcasses of wild birds that died naturally in the field, and 15 fecal samples. Morphological and molecular biological identification revealed that these samples involved 49 bird species belonging to 11 orders and 23 families, accounting for 9.6% of the total bird species in the city (approximately 510 bird species have been recorded in Qinhuangdao City).

Nucleic acid was extracted from all collected samples for viral detection, and AIV-positive samples were subjected to PCR typing and sequencing. The results showed that only one H5N1 AIV-positive sample was identified, while all the others were negative for AIV. This positive sample was obtained from a Larus cachinnans, which was found by local villagers along the beach in December 2024. At the time of discovery, the bird exhibited clinical signs of depression, green feces, and inability to fly. It was promptly sent to Yizhan Bird Rescue Center (Beidaihe District, Qinhuangdao city) for assistance but died the following day. The H5N1 subtype AIV strain isolated from the carcass of this *Larus cachinnans* was designated as A/Seagull/Hebei/qhd6/2024.

### 3.2. Complete Genome Analysis and Phylogenetic Tree Construction of A/Seagull/Hebei/qhd6/2024

To clarify the genomic characteristics, evolutionary origin, and potential risks of the A/Seagull/Hebei/qhd6/2024 (hereinafter referred to as qhd6 strain) isolated in this study, complete genome sequencing and phylogenetic analysis were performed. Strain qhd6′ whole genome sequence was submitted to the GISAID database with Accession No. EPI4158285. Phylogenetic analysis showed its HA gene clustered within H5 subtype AIV clade 2.3.4.4b, forming a monophyletic lineage with Japanese H5N1 strains from crows. In contrast, its NA gene and six internal gene segments (PB2, PB1, PA, NP, M, NS) belonged to the Eurasian lineage (Figure S1). Phylogenetic analysis of internal segments also indicated high sequence similarity with H5N1 strains along the EAAF (suggesting a migratory bird origin) and close relationships with strains from the United States, France, Russia, and Japan, implying intercontinental genetic reassortment potential.

**Figure 1:**
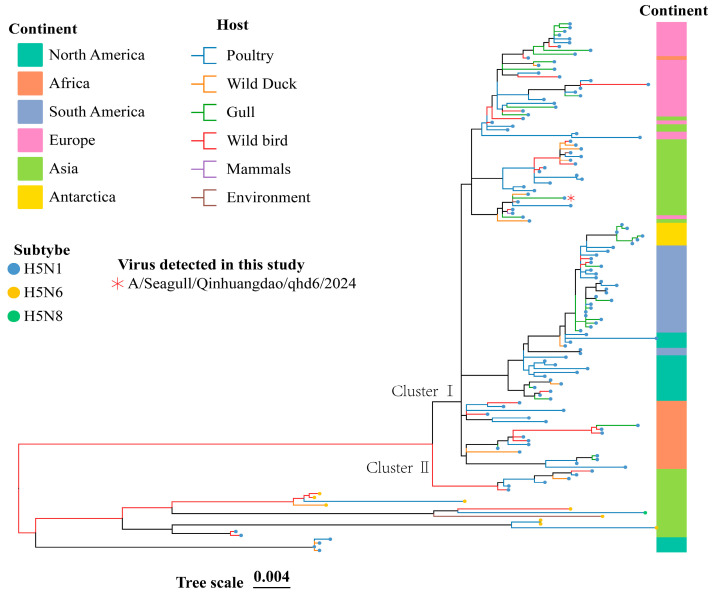
Phylogenetic analysis of the HA gene of contemporary highly pathogenic avian influenza H5 viruses. The maximum-likelihood phylogenetic tree was reconstructed using the best-fit nucleotide substitution model (TIM + F + G4) as determined by IQ-TREE. The analyzed strains encompass the predominant circulating genotypes H5N1, H5N6, and H5N8.

To further elucidate the evolutionary landscape of contemporary HPAIV H5, we performed a comprehensive phylogenetic analysis of the HA gene sequences derived from 139 representative strains documented globally between 2022 and 2024. This curated dataset encompassed the predominant circulating genotypes, including H5N1, H5N6, and H5N8. Maximum-likelihood phylogenetic reconstruction revealed a distinct bifurcation, segregating the viral sequences into two well-supported, deeply divergent monophyletic clades, designated herein as Cluster I and Cluster II. Notably, the isolate qhd6 was unequivocally assigned to the Cluster I lineage (Figure 1). Its phylogenetic placement indicates a close genealogical affinity with a specific subset of contemporary strains, whereas Cluster II likely represents a viral population that is both antigenically and phylogenetically distinct.

**Figure 2:**
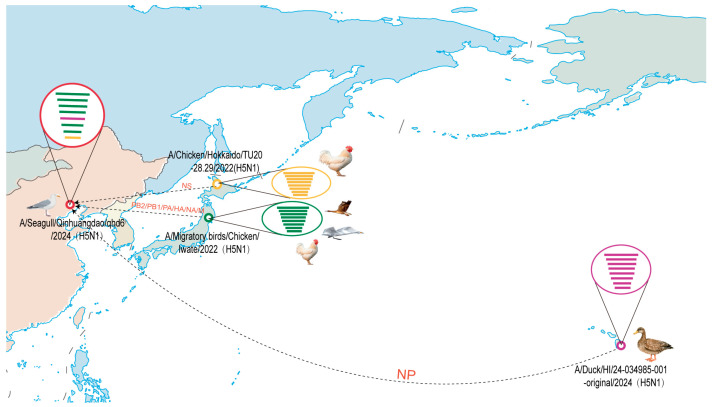
Genetic recombination map of clade 2.3.4.4b H5N1 AIV. Dotted lines in the figure indicate the origins of different gene segments. In the figure, distinct colors indicate different gene segments. From top to bottom, green corresponds to PB2, PB1, PA, HA, NA and M, respectively; purple corresponds to NP; and yellow corresponds to NS. The map is sourced from the National Platform for Common GeoSpatial Information Services. Map approval number: GS(2016)1663.

To confirm its evolutionary origin, NCBI BLAST (2.17.0) analysis of strain qhd6’s full-length genome verified its recombinant nature (Figure 2). Nucleotide identity analysis showed its HA gene shared 99.26% identity with Japanese isolate A/jungle crow/Iwate/0303I003/2022(H5N1), NA gene 99.10% with American isolate A/Mallard/Tennessee/767/2025(H5N1), and internal segments high similarity to strains from the United States, Canada, and Russia (Table 1). These results suggest that the qhd6 HPAI strain may have originated from reassortment of geographically diverse H5 HPAI viruses, consistent with possible intercontinental reassortment.

To evaluate the potential cross-species risk of strain qhd6, its eight gene segments were compared with those of the human-derived H5N1 isolate A/Jiangsu/NJ210/2023. The results showed that the nucleotide identity between the two strains ranged from 90.10% (PB2 gene) to 98.10% (HA gene) (Table 2), suggesting a certain genetic relatedness between strain qhd6 and human-derived H5N1 strains.

### 3.3. Key Amino Acid Mutations of A/Seagull/Hebei/qhd6/2024

To further characterize the HPAI strain following the genomic and recombination analyses described above, we summarized key amino acid mutations (Table 3). Notably, the HA cleavage site of strain qhd6 was PLREKRRKRGLF, consistent with high pathogenicity in wild birds. In addition, a T160A mutation was detected at the HA receptor-binding site (RBS), which has previously been linked to enhanced binding affinity to human-type α2-6 sialic acid receptors and has been associated with increased viral transmissibility in guinea pigs [18].

The polymerase genes of strain qhd6 encode a panel of well-characterized substitutions linked to enhanced viral polymerase activity, including PB2-L89V, G309D, T431M, V598T, PB1-L473V, PA-N383D, and PA-S515T [19,20]. In addition to promoting polymerase activity, these substitutions have been previously linked to elevated pathogenicity in mice and improved replication efficiency in mammalian cells.

Mutations in NA protein- I117T, D199G, I223V, S247N, and H275Y- may contribute to reduced susceptibility to antiviral agents [21,22,23]. Concurrently, mutations in the M1 protein (T215A, N30D, I43M) and NS1 protein (P42S) of strain qhd6 act synergistically to further elevate its pathogenicity in mice [24]. Collectively, these accumulated molecular features have been previously associated with mammalian adaptation and potential cross-species transmission risk, and are present in strain qhd6.

### 3.4. Virus Titer of A/Seagull/Hebei/qhd6/2024 in Larus Cachinnans Organs

**Figure 3:**
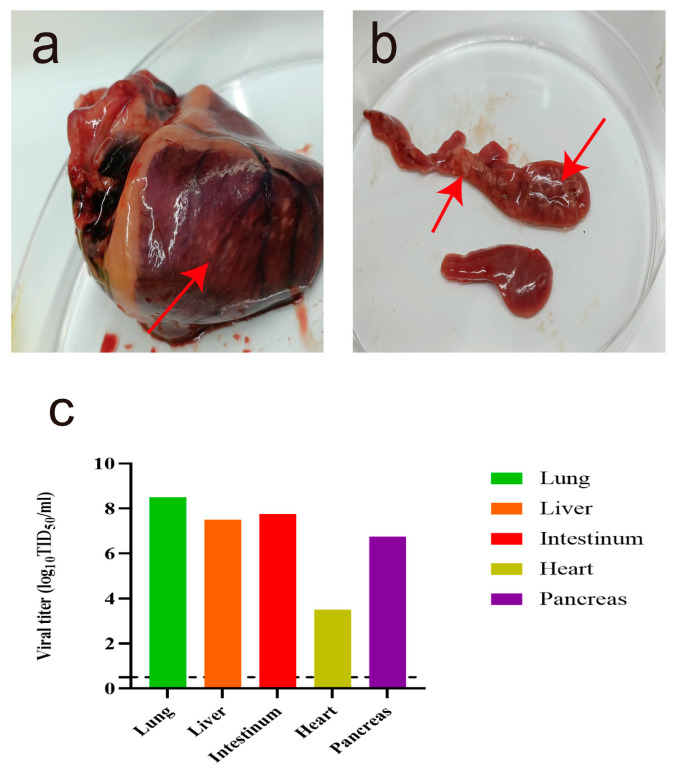
Visceral anatomy and organ viral virulence analysis of *Larus cachinnans*. (**a**) Cardiac anatomical structure. (**b**) Pancreatic anatomical structure. Arrows indicate lesion sites in the corresponding organs. (**c**) Viral loads in tissues collected post-mortem, including lung, liver, intestine, heart, and pancreas.

Gross pathological changes and viral loads were evaluated in *Larus cachinnans* infected with strain qhd6. Pathological dissection revealed prominent lesions in the heart and pancreas, characterized by white nodules of varying sizes, whereas no significant abnormalities were observed in other organs (Figure 3a,b). These manifestations differed from the bronchitis symptoms reported in H5N1-infected gulls by Sheikh et al, indicating that H5N1 subtype AIV may induce distinct clinical signs in seabirds, potentially associated with specific amino acid mutations.

Viral load detection in infected seagull organs showed marked differences among tissues: the lung had the highest viral titer (up to 8.5 TID_50_/mL), while the heart had the lowest (only 3.5 TID_50_/mL). Notably, the pancreas, which showed gross pathological lesions, also had a high viral load (6.75 TID_50_/mL) (Figure 3c), suggesting a possible correlation between viral replication and tissue damage, although histopathological confirmation was not available.

### 3.5. Replication and Virulence of A/Seagull/Hebei/qhd6/2024 in Mice

**Figure 4:**
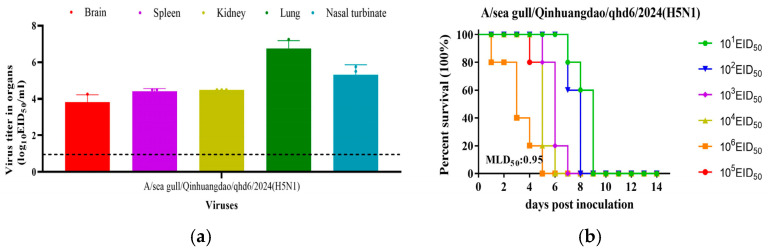
Replication and virulence of qhd6 virus in mice. (**a**) Viral titres in organs of mice that were euthanized on day 3 post-inoculation with 10^6^ EID_50_ of the test virus; (**b**) MLD_50_ of test virus.

To further assess the replicative capacity and virulence of strain qhd6 in mammals, challenge experiments were conducted using BALB/c mice. The virus exhibited systemic replication in infected mice, with viral nucleic acid successfully detected in multiple organs including nasal turbinates, lungs, brain, spleen, and kidneys (Figure 4a). The 50% lethal dose (LD_50_) of strain qhd6 in BALB/c mice was determined to be 0.95 log_10_EID_50_ (Figure 4b), which is consistent with high pathogenicity in BALB/c mice, though this model is inherently susceptible and the finding should be interpreted cautiously.

### 3.6. Cross-Reactivity of A/Seagull/Hebei/qhd6/2024 with Antisera Induced by Different Vaccine Seed Viruses

**Figure 5:**
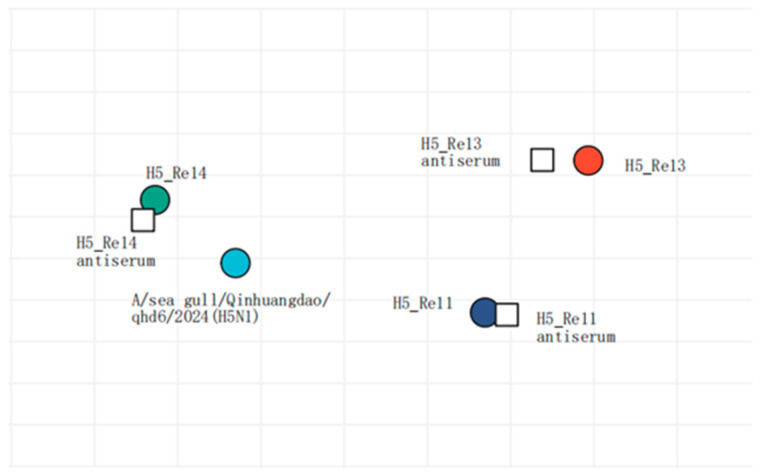
Antigenic cartography of qhd6 virus. The antigenic map was constructed using HI data presented in Table S1. Each unit on the coordinate represents a 2-fold difference in HI titer. Squares represent the antisera generated from the indicated viruses. Different coloured ovals show the viruses used for antisera generation and the test viruses (qhd6).

To evaluate the cross-reactivity of antisera induced by 2018–2022 vaccine seed viruses against strain qhd6, hemagglutination inhibition (HI) assays were performed. The HI antibody titers of H5-Re11, H5-Re13, and H5-Re14 antisera against their homologous viruses were 256, 512, and 512, respectively. Against strain qhd6, H5-Re11 and H5-Re13 antisera showed HI titers of 32 (8-fold and 16-fold lower than their homologous titers, respectively). In contrast, H5-Re14 antiserum exhibited an HI titer of 256 against strain qhd6, only 2-fold lower than its homologous titer. These results demonstrate strong cross-reactivity between H5-Re14-induced antisera and strain qhd6, suggesting that the currently used H5-Re14-based vaccine may provide adequate protection against this virus in poultry (Table S1, Figure 5).

## 4. Discussion

Under the Chinese government’s continuous migratory bird pathogen surveillance, this study conducted targeted AIV monitoring in wild birds at Beidaihe National Wetland Park (a key EAAF node) in Qinhuangdao, a major Bohai Rim coastal port and tourist city. Given AIV’s zoonotic nature, surveillance here is critical. Two-year monitoring (2022–2024) resulted in the isolation of a highly pathogenic H5N1 strain (qhd6) from this wetland; phylogenetic analysis suggests it represents an intercontinental reassortant. Characterization of strain qhd6 further demonstrated its virulence and systemic replication in a mammalian model (BALB/c mice). Additionally, the protective efficacy of current mainstream vaccines against its infection in poultry was evaluated, complementing local epidemiological data and advancing our understanding of H5N1 evolution.

Genetic analyses revealed that the HA gene of strain qhd6 belongs to the globally dominant H5 clade 2.3.4.4b, sharing high sequence homology with H5N1 strains circulating in the EAAF. When compared with H5N1 strains isolated from neighboring regions, including G1 genotype strains from Northeast China and G9 genotype strains from East China [25], strain qhd6 exhibits unique genetic features in its NA gene: it shares high nucleotide identity with North American H5N1 strains, whereas the majority of clade 2.3.4.4b strains in East Asia retain NA genes belonging to the Eurasian lineage. This suggests that strain qhd6 may represent a novel reassortant that has not been commonly detected in East Asian migratory birds, pointing to the considerable genetic diversity of H5N1 along the EAAF. The close phylogenetic relatedness between strain qhd6 and viruses from multiple continents suggests that migratory birds act as key vectors for the dissemination and likely reassortment of H5N1, facilitating genetic exchange among geographically isolated viral populations. Notably, recent global HPAIV infections in cattle also belong to the same clade 2.3.4.4b as strain qhd6, suggesting a shared evolutionary origin and transmission route. Open-range cattle are frequently exposed to migratory birds, their droppings, or contaminated water. As migratory birds are the main carriers of clade 2.3.4.4b viruses, bird migration is the most likely cause of cattle infections [15,26]. Combined with recent cattle outbreaks of the same clade, our results highlight the critical role of migratory birds in the intercontinental and interspecies transmission of clade 2.3.4.4b HPAIVs. This represents a major threat to animal and public health, requiring strengthened surveillance and intervention.

Molecular characterization of strain qhd6 identified multiple adaptive mutations with critical evolutionary significance: HA-T160A mutation enhances binding affinity to human-type α2-6 sialic acid receptors [18], a key step in interspecies transmission; polymerase gene mutations (PB2-L89V/G309D/T431M/V598T, PB1-L473V, PA-N383D/S515T) boost viral replication efficiency in mammalian cells [19,20]; NA-I117T increases potential resistance to neuraminidase inhibitors [21]; and M1 (T215A/N30D/I43M) with NS1-P42S mutations synergistically enhance pathogenicity in mice [24]. These mutations have been previously tied to enhanced mammalian adaptation in other studies. Strain qhd6 exhibited systemic replication in the brain, spleen, kidney, lung, and nasal turbinate, as well as pathogenicity in BALB/c mice (LD_50_ = 0.95 log_10_ EID_50_)—a virulence level comparable to clade 2.3.4.4b H5N1 strains isolated from Europe and North America [25]. Additionally, *Larus cachinnans*—the natural host—infected with strain qhd6 exhibited white nodules in the heart and pancreas, without apparent respiratory lesions. Viral load quantification revealed a gradient of tissue tropism: lung (8.5 TID_50_/mL) > pancreas (6.75 TID_50_/mL) > heart (3.5 TID_50_/mL). The relatively high viral load in the pancreas indicates a tropism for this organ, which differs from most previous studies where pancreatic viral loads were typically lower than those in respiratory tissues. For instance, a 2018 study on H5N1-infected wild ducks reported that respiratory viral titers were significantly higher than pancreatic titers [27], and a 2025 report confirmed lower pancreatic viral loads relative to respiratory tissues in H5N1-infected avian species [28]. These findings suggest a distinctive host immune response and replication pattern of strain qhd6. The molecular mechanism underlying its pancreatic tropism remains unclear, warranting further investigations into virus–host interactions.

HI [29] assay assessed the cross-protective efficacy of H5 vaccine antisera (H5-Re11/13/14) against qhd6. H5-Re11 and H5-Re13 antisera showed low HI titers (32), 8-16-fold lower than their homologous titers (256/512), indicating insufficient cross-protection. In contrast, H5-Re14 antisera maintained a high titer (256), only 2-fold lower than its homologous titer (512), reflecting strong cross-reactivity. With ≥95% HA identity in critical antigenic domains [30] and meeting the protective HI threshold (≥128), H5-Re14 adequately protects poultry. This supports its application for avian influenza control in the Bohai Rim, mitigates threats to the local poultry industry, and emphasizes selecting vaccines matching circulating strains, with future updates prioritizing H5-Re14-like strains.

During the two-year surveillance period, only a single H5N1 strain (qhd6) was isolated, indicating a low detection rate in this wetland ecosystem. This low hit rate may suggest that HPAIV circulate at low levels or are only sporadically introduced into the site, rather than being enzootic in the local bird population. Several factors likely contributed to the limited detections. First, the sampling effort may have missed peak viral shedding periods. Second, sampling gaps may have further constrained our ability to detect the virus. In addition, the sample types collected (e.g., cloacal swabs, fecal samples) and the sensitivity of the detection methods (RT-PCR and virus isolation) may have been insufficient to identify infections with low viral loads or intermittent shedding. RNA degradation during sample transport and storage could also have reduced detection efficiency. Therefore, the absence of additional positive detections does not necessarily indicate the true absence of the virus, but rather reflects inherent limitations in surveillance coverage, sampling design, and diagnostic sensitivity.

Migratory bird migration promotes interregional/intercontinental pathogen spread, and qhd6’s isolation highlights the need for continuous large-scale surveillance in Qinhuangdao to clarify AIV dynamics; integrating genomic sequencing and related studies can improve early warning systems. The study’s limitations include the limited wild bird samples (one qhd6 strain), in vitro HI assays (needing poultry challenge studies), unclear pathogenicity mechanisms, few evaluated vaccine strains, and unassessed transmission risks to poultry and humans.

## 5. Conclusions

This study reports the isolation and characterization of strain qhd6 (H5N1 subtype, 2.3.4.4b clade) from Beidaihe Wetland, North China, and demonstrates its replication capacity and virulence in mice. H5-Re14 vaccines could protect poultry against strain qhd6, enriching understanding of H5N1 evolution, supporting Bohai Rim surveillance and guiding vaccine strategies.

## Figures and Tables

**Table 1:** Influenza virus with the highest nucleotide homology to strain qhd6 in NCBI.

Gene	Virus with the Highest Nucleotide Identity	Accession No.	Identity
PB2	A/northern pintail/USA/IZ24_0474/2024(H5N1) (United States)	PV602201.1	99.54%
PB1	A/northern pintail/USA/IZ24_0474/2024(H5N1) (United States)	PV602201.1	99.37%
PA	A/northern pintail/USA/IZ24_0636/2024(H5N1) (United States)	PV602237.1	99.44%
HA	A/jungle crow/Iwate/0303I003/2022(H5N1) (Japan)	LC718234.1	99.26%
NP	A/Turkey Vulture/CA/25-003487-002-original/2025(H5N1) (United States)	PV337845.1	99.60%
NA	A/Mallard/Tennessee/767/2025(H5N1) (United States)	PV946109.1	99.10%
M	A/whooper swan/Iwate/0303B006/2022(H5N1) (Japan)	LC718245.1	99.50%
NS	A/chicken/Magadan/14-7V/2022(H5N1) (Russia)	PV998155.1	99.55%

Abbreviations: PB2, polymerase basic protein 2; PB1, polymerase basic protein 1; PA, polymerase acidic protein; HA, hemagglutinin; NP, nucleoprotein; NA, neuraminidase; M, matrix protein; NS, nonstructural protein.

**Table 2:** Analysis of the nucleotide homology of strain qhd6 and human H5N1 ^a^.

Virus	Similarity of Each Viral Gene Segment to the Human H5N1 Virus (%)							
qhd6	HA	NA	PB2	PB1	PA	NP	M	NS
	98.1	97.3	90.1	95.3	97.8	96.8	97.3	97.9

^a^ The human H5N1 strain is A/Jiangsu/NJ210/2023(H5N1), which was isolated in Jiangsu Province on 10 February 2023.

**Table 3:** Functional amino acids mutations found in strain qhd6 (H5N1).

Gene Segments	Amino Acids Mutation	qhd6
PB2		
Increased viral polymerase activity and enhanced pathogenicity in mice	PB2-L89V	V
	PB2-G309D	D
	PB2-T431M	M
	PB2-V598T	T
PB1		
Increased viral polymerase activity and replication in mammalian cell lines	PB1-L473V	V
PA		
Increased viral polymerase activity and replication in duck and mammalian cell lines	PA-N383D	D
	PA-S515T	T
Enhanced pathogenicity in mice	PA-K185R	R
NA		
Enhancement of viral resistance to drugs	NA-I117T	T
	NA-D199G	G
	NA-I223V	V
	NA-S247N	N
	NA-H275Y	Y
HA		
Enhanced virus binding to human-type receptor α2-6 sialic acid and increased transmission in guinea pigs	HA-T160A(H3 numbering)	A
M		
Enhanced pathogenicity in mice	M1-T215A	I
	M1-N30D	D
	M1-I43M	M
NS		
Enhanced pathogenicity in mice	NS1-P42S	S

## Data Availability

The original contributions presented in this study are included in the article.

## Supplementary Materials

The following supporting information can be downloaded at: https://www.mdpi.com/article/10.3390/v18060595/s1, Table S1: Cross-reactive HI antibody titers of strain qhd6 (H5N1) with antisera induced by different vaccine seed viruses. Table S2: Reference sequence used in Figure 1. Figure S1: Phylogenetic analysis of HA, NA and internal genes of H5N1 AIV was performed using the maximum likelihood method. The H5N1 virus isolated in this study is marked with red filled circles.
