# Supplementary material for: Clade 2.3.4.4b H5N1 HPAIV from Migratory Birds in Beidaihe Wetland, North China
Source: Viruses. 2026 May 25;18(6):595. doi: 10.3390/v18060595 (PMC13307734; doi:10.3390/v18060595)
Supplement: Supplementary file 1 [file viruses-18-00595-s001.zip › Table S1.pdf]

Table S1. Cross-reactive HI antibody titers of strain qhd6 (H5N1) with antisera induced by different vaccine seed viruses

**Virus**	**Clade**	**HI antibody titer of antiserum induced by different antigens**		
		**H5-Re11**	**H5-Re13**	**H5-Re14**
H5-Re11	2.3.4.4h	256	128	32
H5-Re13	2.3.4.4h	64	512	8
H5-Re14	2.3.4.4b	8	32	512
A/seagull/Qinhuangdao/qhd6/2024(H5N1)	2.3.4.4b	32	32	256
