# Supplementary figures and images for: Clade 2.3.4.4b H5N1 HPAIV from Migratory Birds in Beidaihe Wetland, North China
Source: Viruses. 2026 May 25;18(6):595. doi: 10.3390/v18060595 (PMC13307734; doi:10.3390/v18060595)

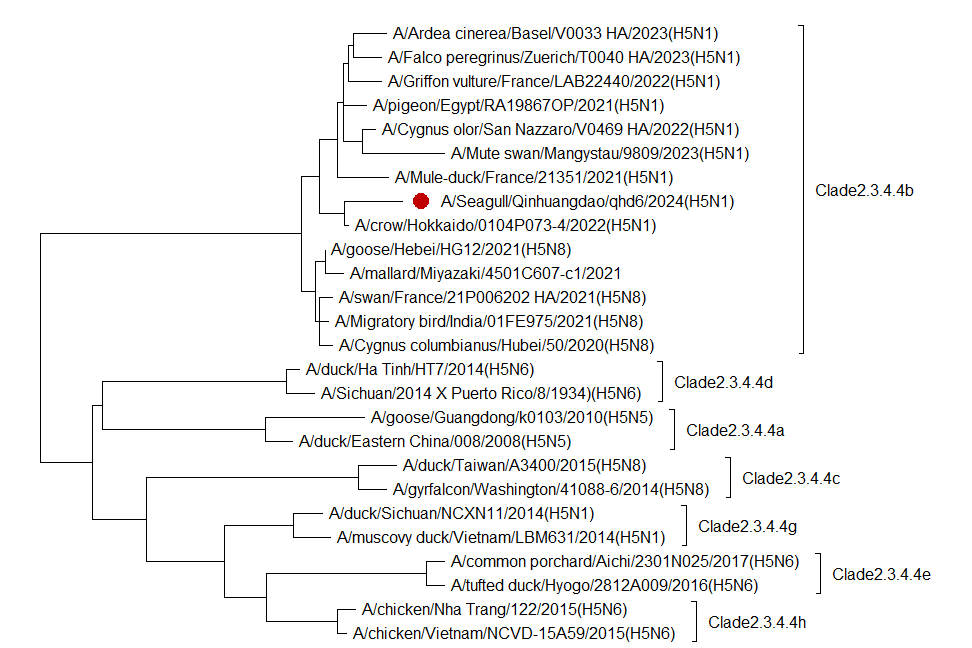

Supplement: Supplementary file 1 [file viruses-18-00595-s001.zip › Figure S1/HA-Clade.png]

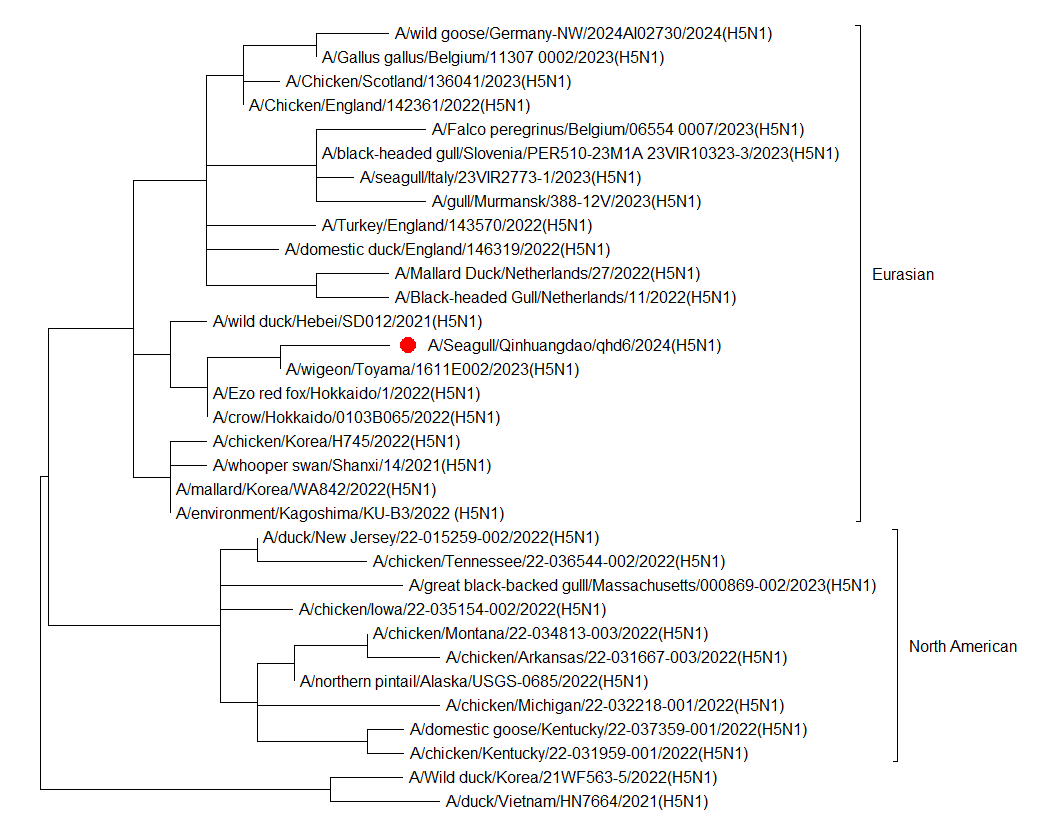

Supplement: Supplementary file 1 [file viruses-18-00595-s001.zip › Figure S1/MP.png]

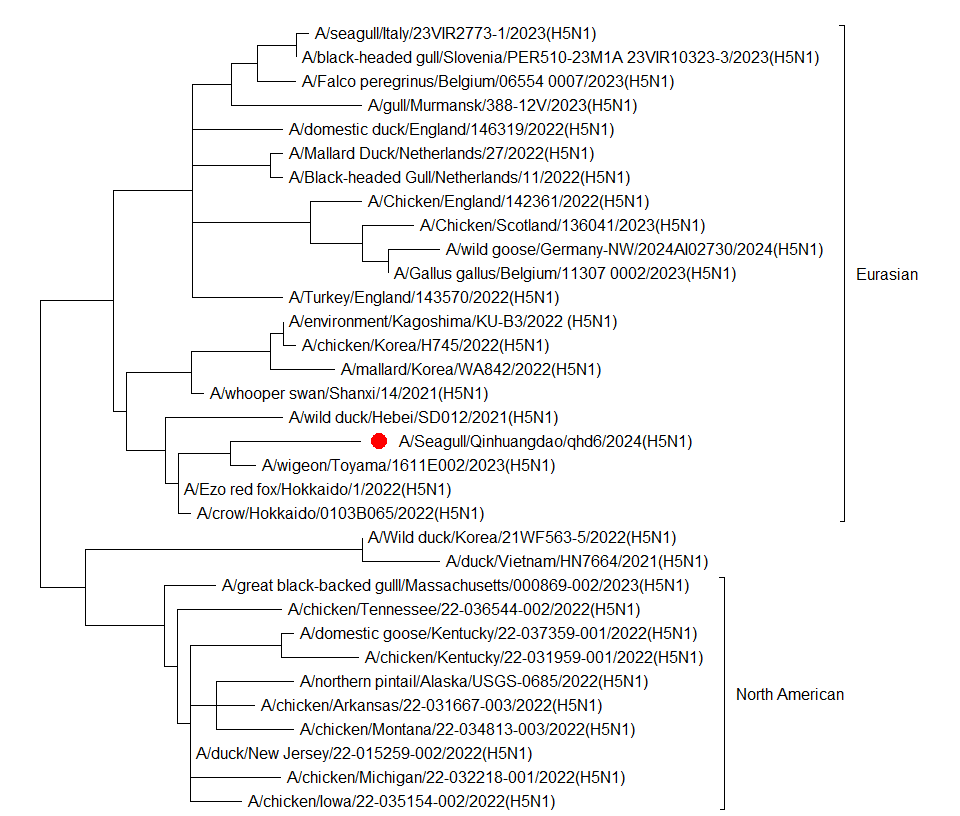

Supplement: Supplementary file 1 [file viruses-18-00595-s001.zip › Figure S1/NA.png]

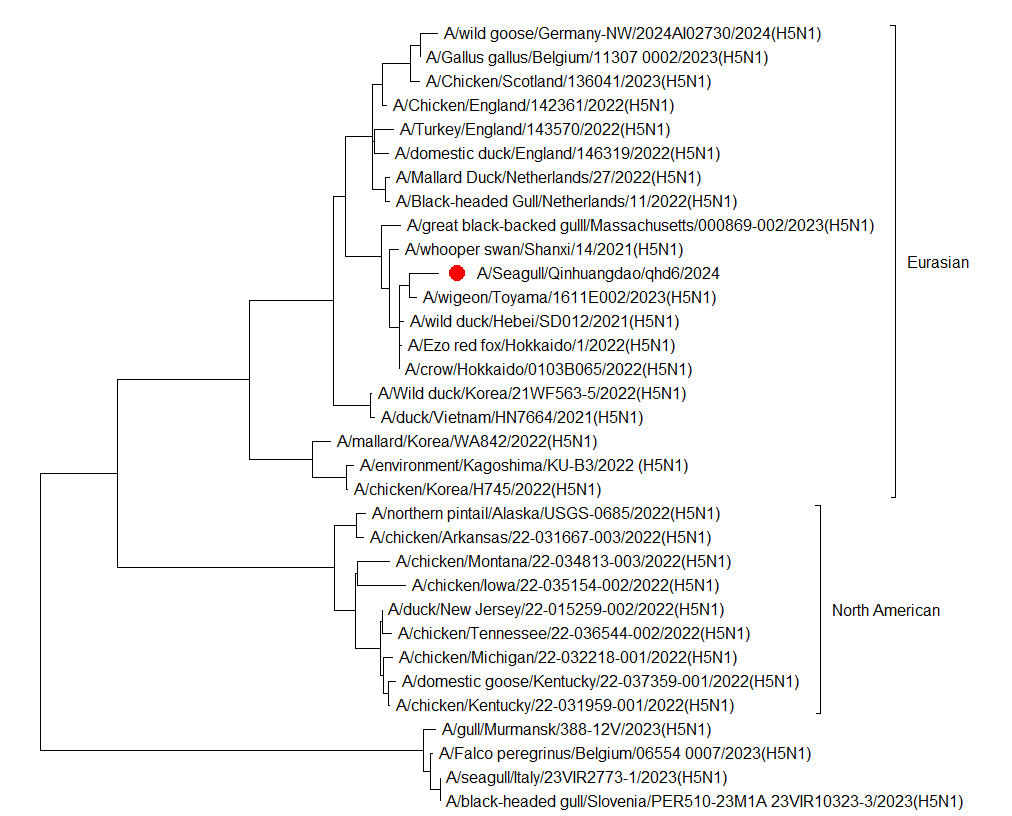

Supplement: Supplementary file 1 [file viruses-18-00595-s001.zip › Figure S1/NP.png]

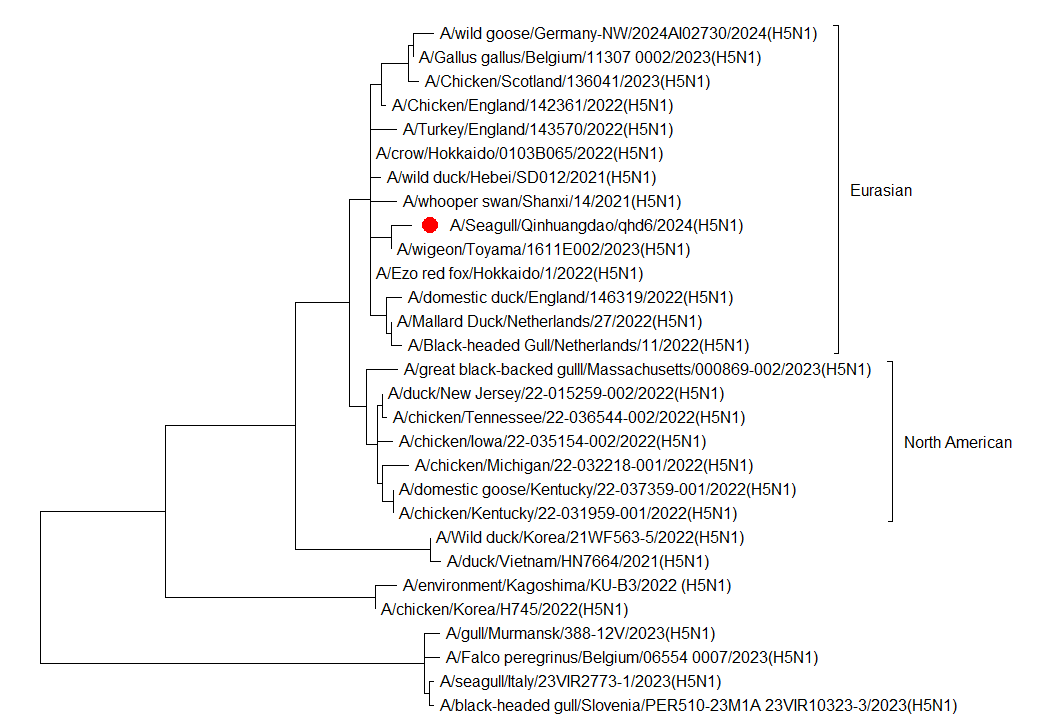

Supplement: Supplementary file 1 [file viruses-18-00595-s001.zip › Figure S1/NS.png]

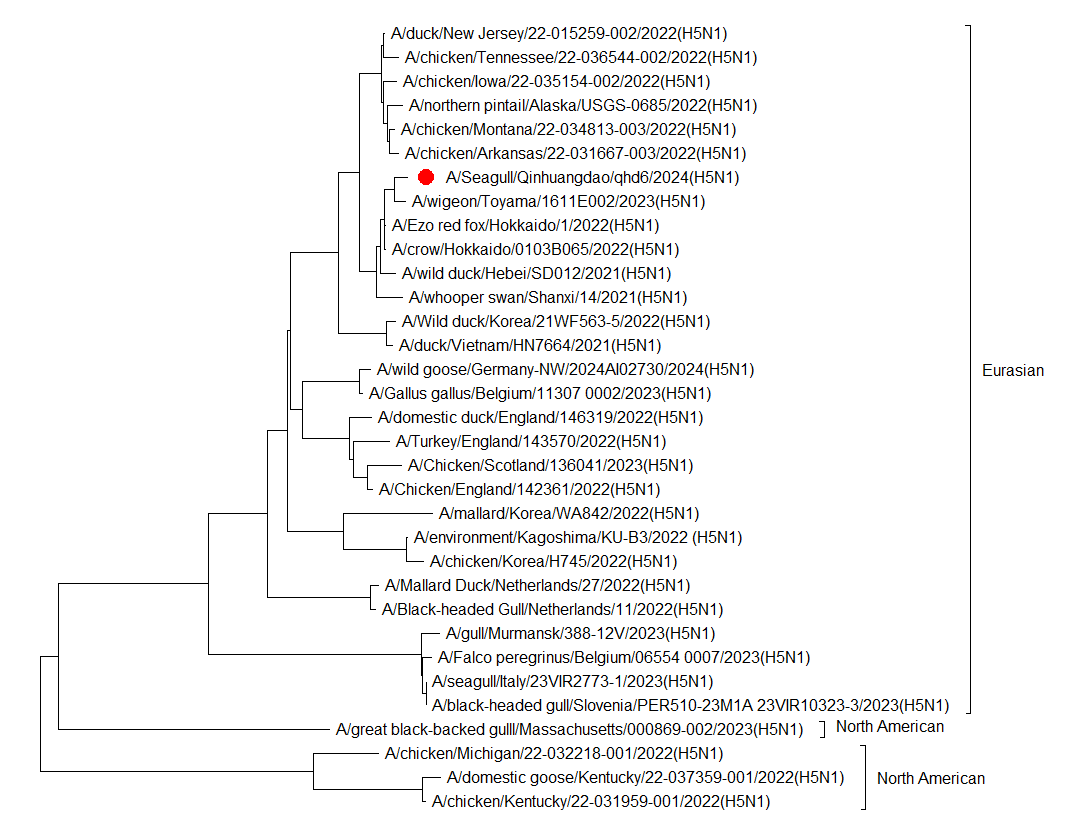

Supplement: Supplementary file 1 [file viruses-18-00595-s001.zip › Figure S1/PA.png]

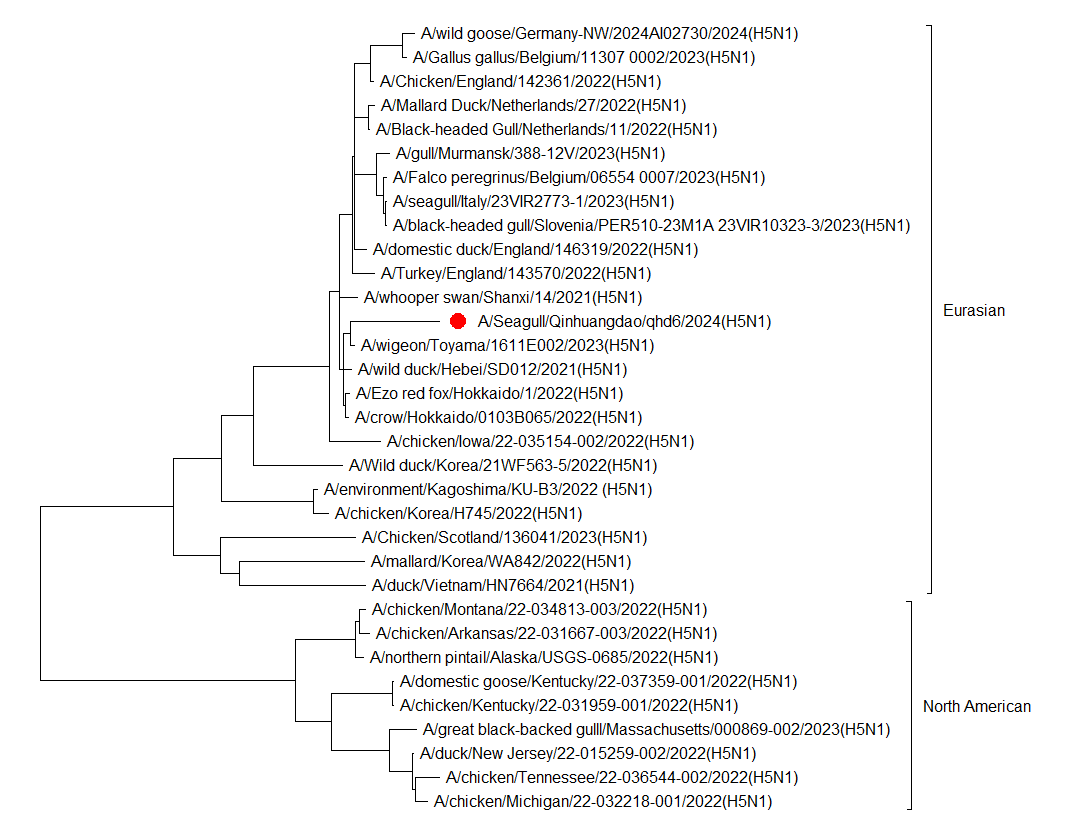

Supplement: Supplementary file 1 [file viruses-18-00595-s001.zip › Figure S1/PB1.png]

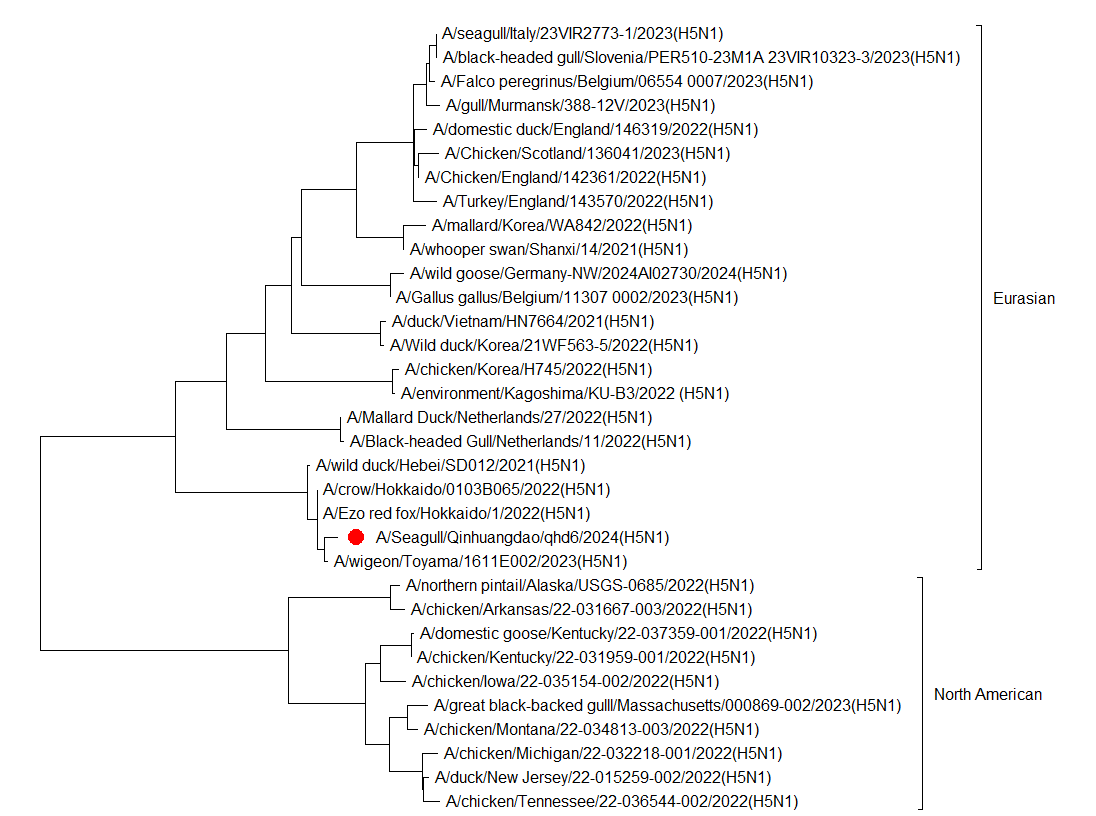

Supplement: Supplementary file 1 [file viruses-18-00595-s001.zip › Figure S1/PB2.png]
